# Renal resistive index is associated with acute kidney injury in COVID-19 patients treated in the intensive care unit

**DOI:** 10.1186/s13089-021-00203-z

**Published:** 2021-02-05

**Authors:** Mårten Renberg, Olof Jonmarker, Naima Kilhamn, Claire Rimes-Stigare, Max Bell, Daniel Hertzberg

**Affiliations:** 1 Department of Perioperative Medicine and Intensive Care, Karolinska University Hospital, Solna, 171 76 Stockholm, Sweden; 2 Department of Physiology and Pharmacology, Karolinska Institutet, Stockholm, Sweden; 3 Department of Radiology, Karolinska University Hospital, Huddinge, Stockholm, Sweden

**Keywords:** Renal resistive index, Point-of-care ultrasound, Ultrasonography, Renal Doppler, Acute kidney injury, COVID-19

## Abstract

**Background:**

Renal resistive index (RRI) is a promising tool for the assessment of acute kidney injury (AKI) in critically ill patients in general, but its role and association to AKI among patients with Coronavirus disease 2019 (COVID-19) is not known.

**Objective:**

The aim of this study was to describe the pattern of RRI in relation to AKI in patients with COVID-19 treated in the intensive care unit.

**Methods:**

In this observational cohort study, RRI was measured in COVID-19 patients in six intensive care units at two sites of a Swedish University Hospital. AKI was defined by the creatinine criteria in the Kidney Disease Improving Global Outcomes classification. We investigated the association between RRI and AKI diagnosis, different AKI stages and urine output.

**Results:**

RRI was measured in 51 patients, of which 23 patients (45%) had AKI at the time of measurement. Median RRI in patients with AKI was 0.80 (IQR 0.71–0.85) compared to 0.72 (IQR 0.67–0.78) in patients without AKI (*p* = 0.004). Compared to patients without AKI, RRI was higher in patients with AKI stage 3 (median 0.83, IQR 0.71–0.85, *p* = 0.006) but not in patients with AKI stage 1 (median 0.76, IQR 0.71–0.83, *p* = 0.347) or AKI stage 2 (median 0.79, min/max 0.79/0.80, *n* = 2, *p* = 0.134). RRI was higher in patients with an ongoing AKI episode compared to patients who never developed AKI (median 0.72, IQR 0.69–0.78, *p* = 0.015) or patients who developed AKI but had recovered at the time of measurement (median 0.68, IQR 0.67–0.81, *p* = 0.021). Oliguric patients had higher RRI (median 0.84, IQR 0.83–0.85) compared to non-oliguric patients (median 0.74, IQR 0.69–0.81) (*p* = 0.009). After multivariable adjustment, RRI was independently associated with AKI (OR for 0.01 increments of RRI 1.22, 95% CI 1.07–1.41).

**Conclusions:**

Critically ill COVID-19 patients with AKI have higher RRI compared to those without AKI, and elevated RRI may have a role in identifying severe and oliguric AKI at the bedside in these patients.

## Background

The Coronavirus disease 2019 (COVID-19) pandemic is causing great suffering and is placing strain on health care systems worldwide. Acute kidney injury (AKI) is a common complication in critically ill patients with COVID-19. Initial studies have reported an incidence from 20 to almost 90% among patients admitted to the intensive care unit (ICU) or in need of mechanical ventilation [1–4], of which up to one-third have been treated with renal replacement therapy (RRT) [5, 6]. COVID-19 patients who develop AKI may have a 13-fold increased risk of death compared to those who do not develop AKI [4, 7, 8].

Renal resistive index (RRI) is an ultrasonographic Doppler measurement of flow velocities in intraparenchymal renal arteries. Normal values are around 0.60 [9, 10] with 0.70 considered the upper normal threshold in adults [11]. Elevated RRI has shown promise in early detection and prognostication of AKI in mixed ICU populations [12–16], and the method seems feasible within the scope of point-of-care ultrasonography (POCUS) [17].

As thromboembolism and renal microangiopathy have gained interest as possible mechanisms giving rise to AKI in COVID-19 [18, 19], RRI may show to be an especially helpful tool to guide diagnosis and treatment of AKI in these patients. In a recent case–control study, reduced renal perfusion and substantially elevated RRI were described in ten COVID-19 patients with severe AKI [20]. However, RRI has not been described in larger populations of COVID-19 patients and its role in these patients remains unclear. The aim of this study was to describe the pattern of RRI in relation to AKI in patients with COVID-19 treated in the ICU. We specifically investigated if there was an association between RRI and AKI diagnosis, different AKI stages and urine output.

## Methods

### Study population

This was an observational cohort study conducted in six ICUs designated for COVID-19 patients (COVID-ICUs) at the Karolinska University Hospital, Stockholm, Sweden. Four ICUs were at one of two sites, and two at the other site. On specific dates, patients in each COVID-ICU were screened for participation. Inclusion criteria were infection with Severe Acute Respiratory Syndrome Coronavirus 2 (SARS-CoV-2) detected by a positive reverse transcriptase-polymerase chain reaction taken from upper or lower airways, admission to a COVID-ICU, and age ≥ 18 years. Exclusion criteria were end-stage renal disease (ESRD) before ICU admission, palliative phase of intensive care, ongoing irregular cardiac rhythm or treatment with extracorporeal membrane oxygenation (ECMO).

The study complied with the Declaration of Helsinki and was approved by the Swedish Ethical Review Authority. Requirement for signed informed consent was waived. A printed information sheet was sent to each patient or next of kin with the opportunity to retrospectively withdraw participation.

### Definitions

AKI was defined according to the Kidney Disease Improving Global Outcomes (KDIGO) classification as an increase in serum creatinine concentration (sCr) and categorized into three stages [21]. The highest sCr from ICU admission to the day of RRI measurement was compared to baseline sCr. Baseline sCr was defined as the last known value measured in a disease-free phase before admission. When no previous sCr value existed, hospital admission sCr was used. The AKI stages were defined as the following: stage 1, ≥ 1.5- to 1.9-fold increase or an absolute increase ≥ 26 μmol/l; stage 2, ≥ 2.0- to 2.9-fold increase; stage 3, ≥ 3.0-fold increase or an absolute increase > 354 μmol/l or initiation of RRT. The KDIGO urine output criteria were not used since hourly urine output was not always registered in the medical records. If the sCr elevation occurred more than 7 days before the RRI measurement and its value had returned to < 1.5-fold or < 26 μmol/l higher than from baseline, the patient was evaluated as having recovered from an AKI episode and was classified into the no AKI group. Oliguria at the time of RRI measurement was defined as urine output < 0.5 ml/kg ideal body weight/hour for 24 h regardless of diuretic drug administration [22]. Ideal body weight was calculated using the gender-specific Acute Respiratory Distress Syndrome Network formula [23]. Chronic kidney disease (CKD) was defined as estimated glomerular filtration rate (eGFR) < 60 mL/min/1.73 m^2^, ESRD was defined as eGFR < 15 mL/min/1.73 m^2^, and eGFR was calculated using the Chronic Kidney Disease Epidemiology Collaboration (CKD-EPI) equation [24]. For this, baseline sCr was used when classifying patients as having CKD or ESRD, and a combination of sCr and cystatin C was used when calculating eGFR at the time of RRI measurement. Comorbidities were considered present if documented in the patient’s medical record or if the patient was prescribed medication for the current state. The cardiovascular disease group included patients with cardiac failure, atrial arrhythmia, prior myocardial infarction or prior cardiac surgery.

### RRI measurements

All RRI measurements were performed by one of two operators (MR and OJ). Both operators had more than one year’s clinical experience of the RRI method. For each site a designated ultrasound device with a curvilinear probe of 1.0–6.0 MHz was used (GE Vivid S70N, US and GE Logiq E10, US at the two sites, respectively). The patients were examined in their ICU bed in supine or prone position depending on their respiratory requirements. Both kidneys were examined, and measurements were made on both or the most accessible side since the difference in RRI values between the right and left kidney has been shown to be negligible both in healthy and critically ill patients [9, 12, 17]. After obtaining a complete view of the kidney, color-Doppler was applied to visualize the global organization of intrarenal blood vessels. Pulse waved Doppler at the smallest possible width between 2 and 5 mm was used to measure flow velocities in an interlobular or arcuate artery in the upper, middle and lower kidney pole. The Doppler gain was set to obtain a clear outline of flow waves with minimal background noise. The pulse waved Doppler spectrum was considered optimal when at least three consecutive similar-looking waveforms for each pole were visualized. RRI was calculated for each pole as [(peak systolic velocity–end-diastolic velocity)/peak systolic velocity]. From the pole RRI values, a mean RRI was computed.

### Data collection

The following clinical data were collected for each patient at the time of RRI measurement: hemodynamic parameters, vasopressor requirements, sedatives dose and ventilator settings if mechanically ventilated. Severity of illness was graded on the day of measurement using the Sequential Organ Failure Assessment (SOFA) (originally the Sepsis-related Organ Failure Assessment) score [25]. Information on comorbidities, regular and current medication and laboratory data were collected from medical records.

### Statistical analysis

Patient characteristics and variables are presented using frequencies and percentages for categorical data, and medians with interquartile range (IQR) and minimum/maximum (min/max) values for continuous data. Clinical characteristics of patients with or without AKI were compared using Fisher’s exact test for dichotomous variables and Wilcoxon rank-sum test for continuous variables. Median RRI between different groups were compared using the Wilcoxon rank-sum test. To elucidate potential confounding of the association between RRI and AKI, a multivariable logistic regression model was performed including RRI as a continuous and AKI as a dichotomous variable. Variables in our dataset that were associated with AKI with a *p*-value < 0.2 in bivariate logistic regression were selected for a manual forward selection procedure. Variables with the strongest association with AKI were included first, and variables that no longer were associated with AKI (*p*-value > 0.2) during forward selection were excluded. Remaining variables constituted the final model. Odds ratios (OR) and 95% confidence intervals (CI) were calculated. Goodness-of-fit of the model was assessed using the Hosmer–Lemeshow test. For all analyses a *p*-value < 0.05 was considered significant. The following variables had missing data: hospital admission sCr (*n* = 7 [14%]) and urine output at the day of RRI measurement (*n* = 2 [4%]). Missing data on height (*n* = 1 [2%]) were substituted with the median value according to sex. Statistical analyses were performed using Stata version 15.1 (StataCorp, College Station, US).

## Results

**Fig. 1:**
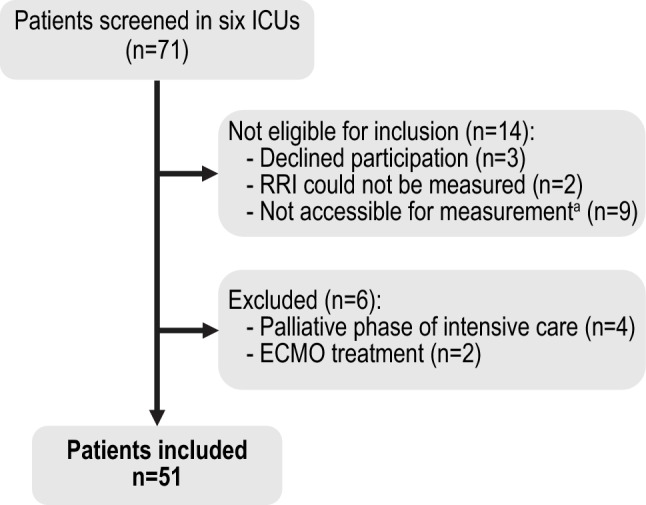
Selection of the study population. ICU, intensive care unit; ECMO, extracorporeal membrane oxygenation. ^a^ Not accessible due to ongoing resuscitation, delirium/agitation, ongoing long period of mobilization/physiotherapy or unknown reason

Between April 15 and May 15 in 2020, the six COVID-ICUs were screened on specific dates for each ICU when at least one of the operators was available and able to perform measurements. Out of 71 screened patients, 20 were excluded, and a total of 51 patients were analyzed (Fig. 1).

Patient characteristics of the study population are presented in Table 1. Median age was 63 (IQR 57–67, min/max 29/74) and 88% were male. At the time of RRI measurement 23 patients (45%) had AKI (stage 1, *n* = 4 (8%); stage 2, *n* = 2 (4%); stage 3, *n* = 17 (33%) with *n* = 13 (25%) treated with continuous RRT [CRRT]). Among the 28 patients (55%) who did not have AKI, 11 patients (22%) previously during the ICU course had an AKI episode but had recovered (recovered from stage 1, *n* = 7 (14%); from stage 2, *n* = 2 (4%); from stage 3, *n* = 2 [4%]) and 17 patients (33%) never had AKI. Compared to patients without AKI, the AKI patients had a higher body mass index (BMI) and a lower incidence of cardiovascular disease. At the time of RRI measurement, AKI patients had a higher SOFA score, a higher incidence of mechanical ventilation and vasopressor use, lower eGFR and were more often oliguric compared to patients without AKI.

**Table 1:** Patient characteristics of all patients and those with and without AKI

	All patients (*n* = 51)	No AKI (*n* = 28)	AKI (*n* = 23)	*p*-value no AKI vs AKI
Age, median (IQR)	63 (57–67)	63 (58–68)	64 (53–65)	0.48
Sex, *n* (%)				0.027
Male	45 (88)	22 (79)	23 (100)	
Female	6 (12)	6 (21)	0 (0)	
BMI, median (IQR)	28.7 (25.2–31.2)	26.8 (24.6–29.9)	30.9 (27.0–34.7)	0.012
Risk factors for AKI, *n* (%)				
Hypertension	29 (57)	15 (54)	14 (61)	0.78
Diabetes	10 (20)	5 (18)	5 (22)	0.74
Chronic lung disease	11 (22)	7 (25)	4 (17)	0.73
Cardiovascular disease	6 (12)	6 (21)	0 (0)	0.027
Chronic kidney disease^a, c^	6 (12)	1 (4)	5 (22)	0.079
End-stage renal disease^b, c^	0 (0)			
No risk factor^d^	15 (29)	7 (25)	8 (35)	0.54
Data at RRI measurement				
ICU day, median (IQR)	18 (6–29)	16 (6–26)	19 (10–31)	0.19
SOFA score, median (IQR)	5 (4–8)	4 (3–7)	7 (5–10)	0.003
Mechanical ventilation, *n* (%)	38 (75)	17 (61)	21 (91)	0.022
Prone position, *n* (%)	9 (18)	5 (18)	4 (17)	1.00
Vasopressors, *n* (%)	26 (51)	10 (36)	16 (70)	0.025
eGFR^c^, median (IQR)	58 (41–75)	70 (52–80)	26 (13–41)	< 0.001
Oliguria^e^, *n* (%)	5 (24)	0 (0)	5 (24)	0.011
RRT, *n* (%)				
Continuous RRT	13 (25)	0 (0)	13 (57)	< 0.001
Intermittent HD	0 (0)			
Treatment in ICU^f^_,_ *n* (%)				
Mechanical ventilation	49 (96)	26 (93)	23 (100)	0.49
Vasopressors	49 (96)	26 (93)	23 (100)	0.49
RRT				
Continuous RRT	13 (25)	0 (0)	13 (57)	< 0.001
Intermittent HD	1 (2)	0 (0)	1 (4)	0.45
Diuretics	48 (94)	25 (89)	23 (100)	0.24
Anti-inflammatory drugs				
Corticosteroids	37 (73)	21 (75)	16 (70)	0.76
Tocilizumab	3 (6)	3 (11)	0 (0)	0.24
Antiviral drugs				
Chloroquine phosphate	1 (2)	0 (0)	1 (4)	0.45
Remdesivir	2 (4)	2 (7)	0 (0)	0.49
Anticoagulation drugs				
LMWH	51 (100)	28 (100)	23 (100)	
Antiplatelets	33 (65)	21 (75)	12 (52)	0.14
Episode of thrombolysis	4 (8)	1 (4)	3 (13)	0.32

*AKI* acute kidney injury, *BMI* body mass index (kg/m^2^), *RRI* renal resistive index, *ICU* intensive care unit, *SOFA score* Sequential Organ Failure Assessment score, *eGFR* estimated glomerular filtration rate (ml/min/1.73 m^2^), *RRT* renal replacement therapy, *HD* hemodialysis, *LMWH* low-molecular-weight heparin

^a^Chronic kidney disease was defined as eGFR before ICU admission < 60 ml/min/1.73 m^2^

^b^End-stage renal disease was defined as eGFR before ICU admission < 15 ml/min/1.73 m^2^

^c^eGFR was calculated using the CKD-EPI equation using serum creatinine for classification of chronic kidney disease and end-stage renal disease, and a combination of serum creatinine and cystatin C at the time of RRI measurement

^d^Patients without history of smoking, chronic diagnoses or regular medications at hospital admission

^e^Oliguria was defined as urine output < 0.5 ml/kg ideal body weight/hour for 24 h

^f^Treatment from ICU admission to RRI measurement

RRI was measured most often in the third week of the ICU and hospital course (median ICU day 18, IQR 6–29, min/max 0/37; median hospital day 20, IQR 10–30, min/max 2/37) and 4 weeks from symptom debut of COVID-19 (median 28, IQR 22–40, min/max 7/72). In 27 patients (53%) RRI was calculated from measurements in the right kidney, in 12 patients (23,5%) from measurements in the left kidney and in 12 patients (23,5%) from measurements in both kidneys. Forty-two patients (82%) were examined in supine and nine patients (18%) in prone position.

### RRI in relation to AKI and AKI stage

**Fig. 2:**
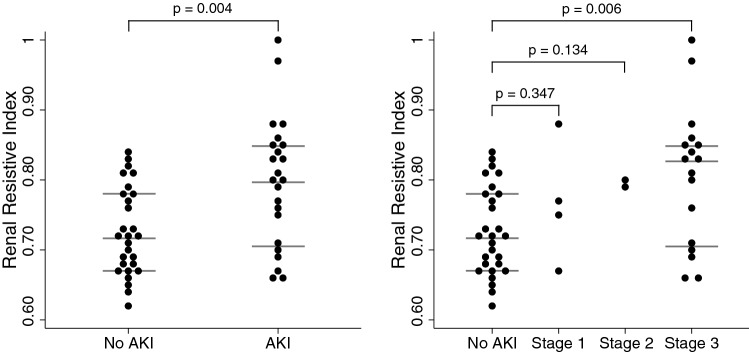
Dot plot illustrating the association between renal resistive index and acute kidney injury (AKI) in patients with or without AKI (left) and patients with different stages of AKI (right). Each dot represents a patient. The horizontal lines represent the median, upper and lower quartiles

Median RRI in the study population was 0.76 (IQR 0.69–0.82, min/max 0.62/1.0). One patient had completely diminished end-diastolic blood flow resulting in an RRI of 1.0. Median RRI in patients with AKI was 0.80 (IQR 0.71–0.85, min/max 0.66/1.0) compared to 0.72 (IQR 0.67–0.78, min/max 0.62/0.84) in patients without AKI (*p* = 0.004) (Fig. 2). There was no difference in RRI between AKI stage 1 (median 0.76, IQR 0.71–0.83, min/max 0.67/0.88) or AKI stage 2 (median 0.79, min/max 0.79/0.80, *n* = 2) compared to no AKI (*p* = 0.347 and 0.134, respectively), but RRI was higher in patients with AKI stage 3 (median 0.83, IQR 0.71–0.85, min/max 0.66/1.0) compared to patients without AKI (*p* = 0.006) (Fig. 2).

### RRI in relation to non-AKI, recovered AKI and ongoing AKI

**Fig. 3:**
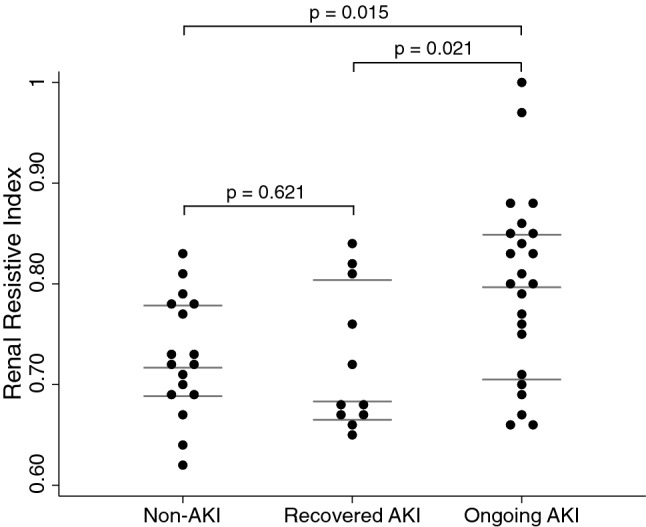
Dot plot illustrating the association between renal resistive index and acute kidney injury (AKI) in patients who did not develop AKI, developed AKI but recovered, and had an ongoing AKI episode at the time of measurement. Each dot represents a patient. The horizontal lines represent the median, upper and lower quartiles

RRI did not differ within the no AKI group when comparing patients who never had AKI (median 0.72, IQR 0.69–0.78, min/max 0.62/0.83) to patients with recovered AKI (median 0.68, IQR 0.67–0.81, min/max 0.65/0.84) (*p* = 0.621), but RRI was higher in the AKI group compared to both these groups (*p* = 0.015 and 0.021, respectively) (Fig. 3).

### RRI in relation to oliguria

**Fig. 4:**
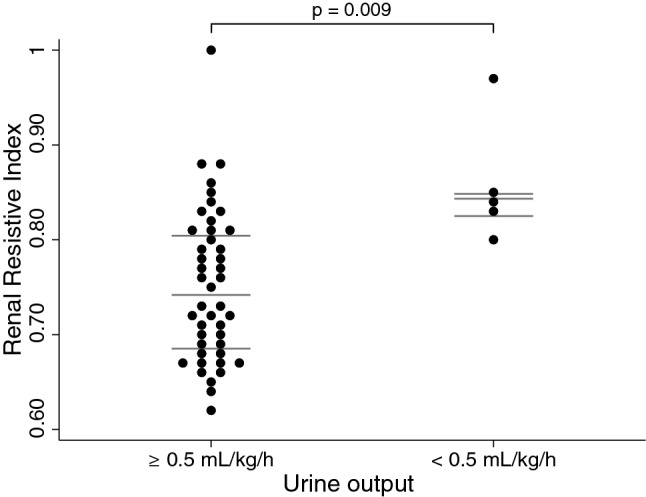
Dot plot illustrating the association between renal resistive index and oliguria defined as urine output < 0.5 ml/kg ideal body weight/hour for 24 h. Each dot represents a patient. The horizontal lines represent the median, upper and lower quartiles

RRI was higher in oliguric patients (median 0.84, IQR 0.83–0.85, min/max 0.80/0.97) compared to non-oliguric patients (median 0.74, IQR 0.69–0.81, min/max 0.62/1.0) (*p* = 0.009) (Fig. 4).

### Multivariable analysis

Bivariate and multivariable logistic regression analysis is presented in Table 2. Possible confounders eligible for inclusion in the multivariable model are all variables presented in Table 1 except those that could be influenced by AKI and hence be part of a causal chain (ICU day, SOFA score, eGFR, oliguria, RRT, administration of diuretics). The following variables were omitted due to perfect prediction in the AKI groups (sex, cardiovascular disease, mechanical ventilation during ICU course, vasopressors during ICU course, tocilizumab, chloroquine phosphate, remdesivir, low-molecular-weight heparin). Variables included in the final model were RRI, BMI, CKD, vasopressors at measurement and administration of antiplatelet drugs. After multivariable adjustment, RRI was independently associated with AKI (OR for 0.01 increments of RRI 1.22, 95% CI 1.07–1.41). Goodness-of-fit had *p* = 0.705.

**Table 2:** Bivariate and multivariable analysis showing the association with AKI

	Bivariate analysis		Multivariable analysis^a^	
	OR (95% CI)	*p*-value	OR (95% CI)	*p*-value
RRI (0.01 unit step)	1.13 (1.04–1.23)	0.005	1.22^b^ (1.07–1.41)	0.004
Age	0.97 (0.91–1.03)	0.296		
BMI	1.11 (0.99–1.23)	0.067	1.23 (1.02–1.48)	0.031
Risk factors for AKI				
Hypertension	1.35 (0.44–4.13)	0.601		
Diabetes	1.28 (0.32–5.10)	0.729		
Chronic lung disease	0.63 (0.16–2.50)	0.513		
Chronic kidney disease^c^	7.50 (0.81–70)	0.076	175 (3.72–8281)	0.009
No risk factor^d^	1.60 (0.48–5.37)	0.447		
Data at RRI measurement				
Mechanical ventilation	6.79 (1.32–35)	0.022	^f^	
Prone position	0.97 (0.23–4.12)	0.965		
Vasopressors	4.11 (1.27–13)	0.019	27 (2.68–268)	0.005
Treatment in ICU^e^				
Anti-inflammatory drugs				
Corticosteroids	0.76 (0.22–2.61)	0.666		
Anticoagulation drugs				
Antiplatelets	0.36 (0.11–1.19)	0.094	0.18 (0.03–1.17)	0.073
Episode of thrombolysis	4.05 (0.39–42)	0.241		

*AKI* acute kidney injury, *OR* odds ratio, *CI* confidence interval, *RRI* renal resistive index, *BMI* body mass index (kg/m^2^), *ICU* intensive care unit

^a^Goodness-of-fit *p* = 0.705

^b^An increase of 0.01 in RRI increased the risk of having AKI at measurement by 22%

^c^Chronic kidney disease was defined as eGFR before ICU admission < 60 ml/min/1.73 m^2^

^d^Patients without history of smoking, chronic diagnoses or regular medications at hospital admission

^e^Treatment from ICU admission to RRI measurement

^f^Variable excluded from multivariable logistic regression model during forward selection

## Discussion

This study presents novel findings of the pattern of RRI in critically ill patients with COVID-19. RRI was higher in patients with AKI compared to patients without AKI, and the difference was significant in patients with AKI stage 3 but not in patients with AKI stage 1 or 2. RRI was higher in patients with an ongoing AKI episode compared to patients who had recovered from AKI earlier during the ICU course. Oliguric patients had higher RRI compared to non-oliguric patients. The association between RRI and AKI remained significant after adjustment for possible confounding.

Our results are in line with previous studies on mixed or septic ICU patients instead designed to investigate the role of RRI to predict and prognosticate AKI [12, 13, 16, 26, 27]. These studies have suggested RRI to be able to distinguish severe or persistent AKI from no or transient AKI, with optimal cut-off values for this discrimination varying from 0.69 to 0.80. The median RRI of 0.80 in patients with AKI in our population must be considered high in comparison, but may partly be due to the large proportion of patients with AKI stage 3. This is in line with the results of a recent case–control study which presented higher RRI in ten COVID-19 patients with AKI stage 3 compared to ten patients with AKI from septic shock [20]. Notably, also patients without AKI in our study had higher RRI (median 0.72) compared to non-AKI patients in ICU populations without COVID-19 where reported values typically are lower than 0.65 [12, 14, 28]. It is not clear if elevated RRI in patients without AKI but infected with SARS-CoV-2 is specifically related to the infection itself, or if it reflects severity of illness as indicated by the long length of ICU stay as well as the high incidence of mechanical ventilation and vasopressor use in our population.

There is growing evidence of reduced renal microperfusion in patients with COVID-19-related AKI, both from contrast enhanced ultrasonographic measurements in patients with severe AKI [20] as well as from post-mortem findings of microvascular obstruction [29]. In addition, numerous studies have reported high rates of thrombotic complications in hospitalized COVID-19 patients in general [30–32], and thrombi in the renal microcirculation has been mentioned as a possible mechanism contributing to AKI in these patients [2]. Even if several renal and extrarenal factors influence the final profile of the RRI flow wave and value [33], it is conceivable that renal microcirculatory disturbances further contributed to the generally high RRI values observed in our population.

Previous studies on critically ill patients without COVID-19 have in general focused on prediction of AKI from RRI measurements performed within the first day of ICU admission [12, 14–16, 26]. However, the ability of early RRI measurements to predict short-term AKI reversibility within 3 days recently has been challenged [34, 35]. Our finding of higher RRI in COVID-19 patients with an ongoing AKI episode compared to patients who had recovered from an AKI episode earlier during the ICU course suggests that RRI values decrease with recovered renal function. This indicates that RRI also might have a role later in the ICU or hospital course, but its exact role for prediction of renal recovery or progression towards CKD while the patient still is in hospital needs to be investigated in properly designed studies. In outpatients with already established CKD, RRI ≥ 0.70 notably has shown to be predictive of both CKD progression [36, 37] and mortality [38].

Our finding of elevated RRI in COVID-19-related AKI indicates that RRI may have a role in the assessment of this new and unique disease. Further, RRI has shown potential as a precocious ICU monitoring tool for detecting progression of shock states [39, 40], and together with Doppler assessments of other splanchnic organs RRI can expand the bedside monitoring window for hypoperfusion in critically ill patients in general [41]. As a non-invasive and repeatable method that has been demonstrated to be fast to learn also for non-experienced sonographers [17, 42], RRI should be applicable within POCUS protocols for ICU clinicians and thereby contributing as a valuable tool in the present resource scarce times of a pandemic.

Our study has several limitations. First, our study population was small and although we were still able to adjust our analysis for a number of possible confounders, the results should therefore be interpreted with caution. The small number of patients with AKI stage 1 and 2 further makes it difficult to draw conclusions on the association between RRI and AKI in these specific subgroups. Second, due to the challenges of conducting clinical research during a pandemic, RRI measurements were performed at different time points in different patients and most of the measurements were made late in the ICU course. The Karolinska University Hospital is a tertiary referral hospital, and many patients were transferred to its ICUs from other hospitals when they had already received several days of intensive care. This meant there was a delay from ICU admission to accessibility in some of the patients, and we were therefore not able to perform early measurements to investigate the ability of RRI as an early predictor of subsequent AKI development. Third, intra- and inter-observer variability for the operators were not investigated. Our group has previously shown that RRI measurements by inexperienced sonographers were reliable, accurate and precise compared to an expert after only a brief training session [17], and both operators in our study were experienced with the RRI method. Lastly, our study was affected by some of the well-known pitfalls in AKI research. The use of hospital admission sCr as baseline level in patients in whom pre-admission sCr was missing might have resulted in an underestimation of the AKI incidence. Further, using sCr decline to define recovery from an AKI episode could in patients with muscle wasting during a prolonged ICU course lead to overestimation of renal function recovery [43]. However, we used eGFR calculations based on a combination of sCr and cystatin C at the time of RRI measurement and still observed a difference in estimated renal function between patients classified with or without AKI, suggesting any such misclassification was negligible.

## Conclusion

Critically ill COVID-19 patients with AKI have higher RRI compared to those without AKI, and elevated RRI may have a role in identifying severe and oliguric AKI at the bedside in these patients. The exact role of RRI as a POCUS application for AKI assessment and monitoring of ICU patients with COVID-19 should be established in further studies.

## Data Availability

The datasets used and analyzed during the current study are available from the corresponding author on reasonable request.

## Abbreviations

COVID-19: Coronavirus disease 2019
AKI: Acute kidney injury
ICU: Intensive care unit
RRT: Renal replacement therapy
RRI: Renal resistive index
POCUS: Point-of-care ultrasonography
COVID-ICU: Intensive care unit designated for care of COVID-19 patients
SARS-CoV-2: Severe acute respiratory syndrome coronavirus 2
ESRD: End-stage renal disease
ECMO: Extracorporeal membrane oxygenation
KDIGO: Kidney Disease Improving Global Outcomes
sCr: Serum creatinine concentration
eGFR: Estimated glomerular filtration rate
CKD-EPI: Chronic Kidney Disease Epidemiology Collaboration
CKD: Chronic kidney disease
SOFA: Sequential Organ Failure Assessment
IQR: Interquartile range
min/max: Minimum/maximum
OR: Odds ratio
CI: Confidence interval
CRRT: Continuous renal replacement therapy
BMI: Body mass index
